# Supplementary material for: Video-rate multi-color structured illumination microscopy with simultaneous real-time reconstruction
Source: Nat Commun. 2019 Sep 20;10:4315. doi: 10.1038/s41467-019-12165-x (PMC6754501; doi:10.1038/s41467-019-12165-x)
Supplement: Supplementary file 1 — Supplementary Information [file 41467_2019_12165_MOESM1_ESM.pdf]

Video-rate multi-color structured illumination microscopy with simultaneous real-time reconstruction

Supplementary Information

Markwirth et al.

Supplementary Note 1: Timing of ferro-electric SLMs and sCMOS cameras

The ferro-electric SLM

Working principle

The ferro-electric spatial light modulator is a central component in many recent SLM-based SIM setups. Its reflective pixels act as an electronically controllable wave plate, which can be toggled between its two binary states. The polarization change introduced by the pixels allow to use the SLM as a switchable optical phase grating:

All pixels always reflect light, but depending on their on/off state, their fast axis differs by 33°, so they rotate the polarization of the reflected light by a different angle. For light with a wavelength of 555nm, the angle between these two polarizations is approximately 66°.

To describe this polarization-based interference effect, an orthogonal base is chosen, with the angle bisector used as one coordinate axis. This base vector describes the portion of the light that has the same (parallel) polarization for both angles, while the orthogonal component is obviously polarized anti-parallel. The parallel components of the polarization of the reflected beams are the same for pixels in both on- and off-states and form the 0th order maximum.

The anti-parallel components could just as well be described as having the same polarization, but a 180° phase-offset to each other, depending on the on/off state, resulting in an interference-pattern. Therefore, the SLM can be understood as an electronically controlled binary optical phase grating.

Timing constrains:

Binary patterns (resolution 1280×1024 pixels) are uploaded to the device through manufacturer-provided software, and can be arranged into sequences, so-called "running orders", in which timings and break points (waiting for a TTL-pulse) can be defined.

We found the following characteristics helpful to quickly asses the capabilities and limitations of the device:

- Each pattern is displayed for a fixed time, selectable from a manufacturer-provided list. Available timings in the range relevant for fast SIM are 0.5 ms, 1 ms, 2 ms, and 5 ms. Intermediate timings could be obtained from the manufacturer at additional cost.
- Due to charge-build-up in the display, the inverse of each image in a sequence has to be displayed. Timing requirements are such that the inverse of each image has to finish displaying within 50 ms of the start of the positive image display.
- Switching between patterns takes approximately 0.44 ms, during which pixels are undefined, so no illumination should take place.
- Delays of arbitrary but defined length can be inserted in the switching cycle between images, as long as the inverse images are still displayed within 50 ms. During delays, the SLM pixels stay undefined.
- The device is in a balanced state, when for every image its inverse has finished displaying.
- Pauses of undefined length, i.e., waiting for external synchronization triggers, can only be inserted at points where the display is balanced.

Under these constraints, a few combinations of SIM image acquisitions can be implemented. If three wavelengths are to be used, 27 raw images (3 angles × 3 phases × 3 colors) have to be acquired. This sequence can be completed within the limit of 50ms if 1ms exposure time is used. Illuminating with 2 wavelengths, the required 18 raw images (3 angles × 3 phases × 2 colors) fit the 50ms constraint when using either 1ms or 2ms illumination per frame. In the former case, the afore mentioned delays can be added to allow for a full (512×512) camera readout (see below).

The sCMOS cameras

The SLM has to be synchronized with the sCMOS camera systems. A set of pco.edge 4.2 cameras, as well as a Hamamatsu Orca Flash 4.0 (employing the same sCMOS chip), have been tested and used in the system. In both cases, the *Camera Link* variants of the cameras were used with 16 bit depth and the fast readout mode. Other cameras and interface types (e.g. current-generation sCMOS cameras with USB 3.0 and 3.1 interfaces) should –

in principle – also be capable of providing similar speeds to those reached by our system. However, an in-depth understanding of the camera's timing characteristics as well as interface and firmware capabilities might be necessary to ensure compatibility:

Unlike (em-)CCD systems, where speed is typically proportional to the total numbers of pixels to be sampled, the speed of current sCMOS chips depends on the number of lines to be read from the chip. The chip in use here runs at 4.83 µs (PCO) or 4.88 µs (Hamamatsu) per line (for a symmetric ROI, with 2 read-out-circuits at 9.65 µs (PCO) or 9.74 µs (Hamamatsu) per line), which is among the fastest systems currently available. The camera operates in so-called "rolling shutter" mode, i.e., the built-up charge is read directly from the light-sensitive pixels, line by line.

While this yields equal exposure times for each line, the start and end of exposure is different for every line (by the corresponding multiple of the line readout time).A "global shutter" mode, comparable to the shutter of frame transfer (em)CCD sensors, could mitigate this effect, but worsens signal-to-noise and is not available for this camera system.

The effect of the shutter operation has to be considered with respect to the experiment to run (wide-field, localization microscopy, light-sheet). For structured illumination, where the switch in illumination pattern causes a global change in each frame, a simple and robust scheme is to cleanly separate exposure and read-out: The chip is exposed in full, then the light source is turned off and a trigger pulse starts the read-out and reset of the chip. Only after this read-out and reset is complete, the next pattern is illuminated, again to be read out only after the illumination has been turned off again.

It should be noted that this overall illumination and read-out scheme (see Supplementary Figures 1 and 2 for an in-depth example with timing diagrams), especially with its use of a dedicated camera per color channel, yields a setup optimized for the fastest multi-channel acquisition possible. The more classical approach of a *dual-view* or *image-splitting* device, where two or more color channels are imaged onto the same camera chip, would create dead-times whenever that single camera chip needs to be read out, and thus cannot be illuminated. This would be alleviated only by the use of a global shutter camera, or a rolling shutter system with a single read-out direction in line with the image splitting direction. Also, from an economic standpoint, the difference both in cost and flexibility of using two cameras vs. a single camera and an image splitting device, have to be evaluated. Besides speed, two further important camera characteristics for SIM imaging are sensitivity and linearity. The sensitivity of current-generation sCMOS cameras, given by their quantum efficiency and read-noise, is very high, easily reaching levels suitable for single-molecule detection (although single fluorescent molecules typically lack the linear behavior required for SIM due to blinking and bleaching effects). The linearity of the cameras is typically optimized by the manufacturer, as they aim to provide a quantitative imaging tool. Both aspects should, however, be considered when selecting a different camera system, especially when striving for better cost-effectiveness.

Supplementary Figure 1: Timing diagram for the 2 colors, 1 ms mode

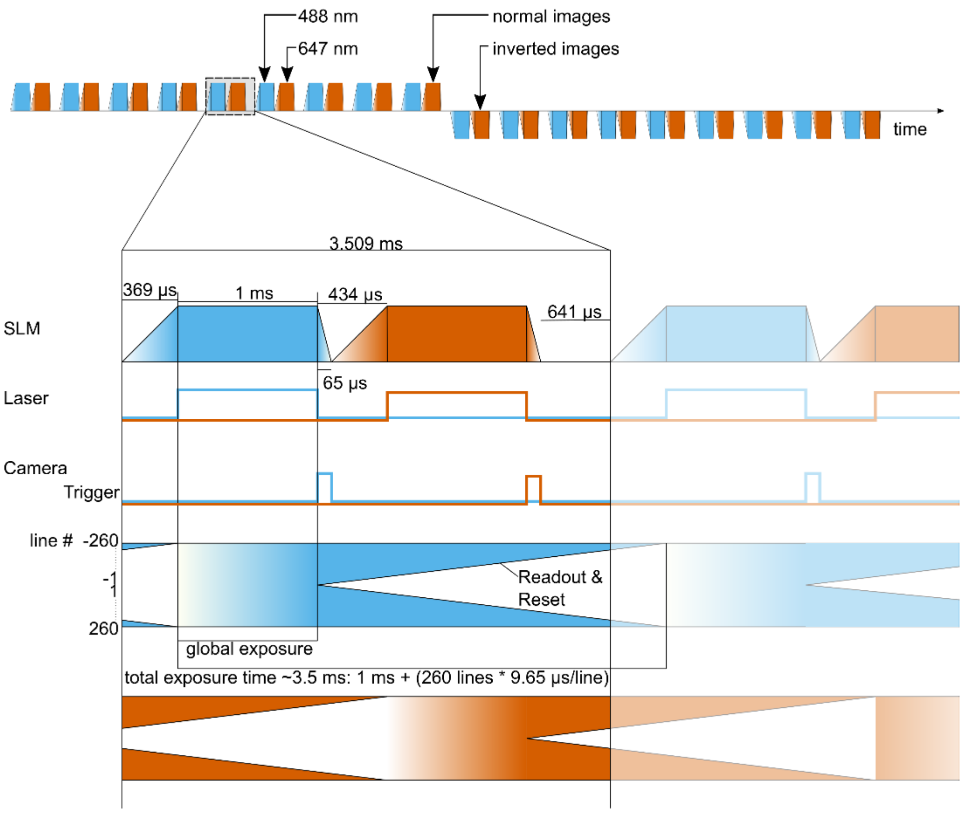

Timing schematic for the 2 color, 1 ms mode with pco.edge 4.2 cameras.

The FLCoS-SLM displays 2×9 individual images for 1 ms each (plus 434 µs switching time), before the inverted images ("on" and "off" pixels are switched) are shown. Both images lead to the same illumination pattern, so both can be used for image acquisition. Both must be displayed within a strict time-limit to avoid damage to the SLM, which is enforced by the manufacturer's software.

The cameras need 9.65 µs to read two lines, symmetric around the center. The number of lines can be set in multiples of 20, so for an image with 512×512 pixels, 520 lines must be read. This takes approximately 2.5 ms.

In our timing scheme, this readout time of one camera is used to take images with the other camera. In the 2-color mode shown here, the illumination and camera readout take longer (3.5 ms) than the 2.9 ms the SLM needs to display two images with 1 ms exposure and 434 µs switching each. In this mode, the readout speed of the cameras limits the acquisition speed of the microscope.

The camera trigger starts the "reset" of the sCMOS camera chip, which removes the collected charge. The cameras exposure time is set to 3.5 ms in MicroManager. With 2.5 ms of readout-time, this leads to 1 ms of global exposure, after which the readout starts automatically. The camera then waits for the next trigger to start the next reset-sequence.

The laser lines are only turned on after all 520 lines of their respective cameras have been reset and are turned off when the readout starts (global exposure).

The timing is carefully adjusted on the microcontroller: A digital storage oscilloscope is used to monitor the laser and trigger signals from the microcontroller, the "trigger ready" and exposure-output signals of the cameras and the LED-enable output of the SLM. The initial timings used in the microcontroller software, which can be calculated from the graph shown above, are adjusted to the real-world conditions, with a few microseconds to spare to allow for some jitter.

Supplementary Figure 2: Timing diagram for the 3 colors, 1 ms mode

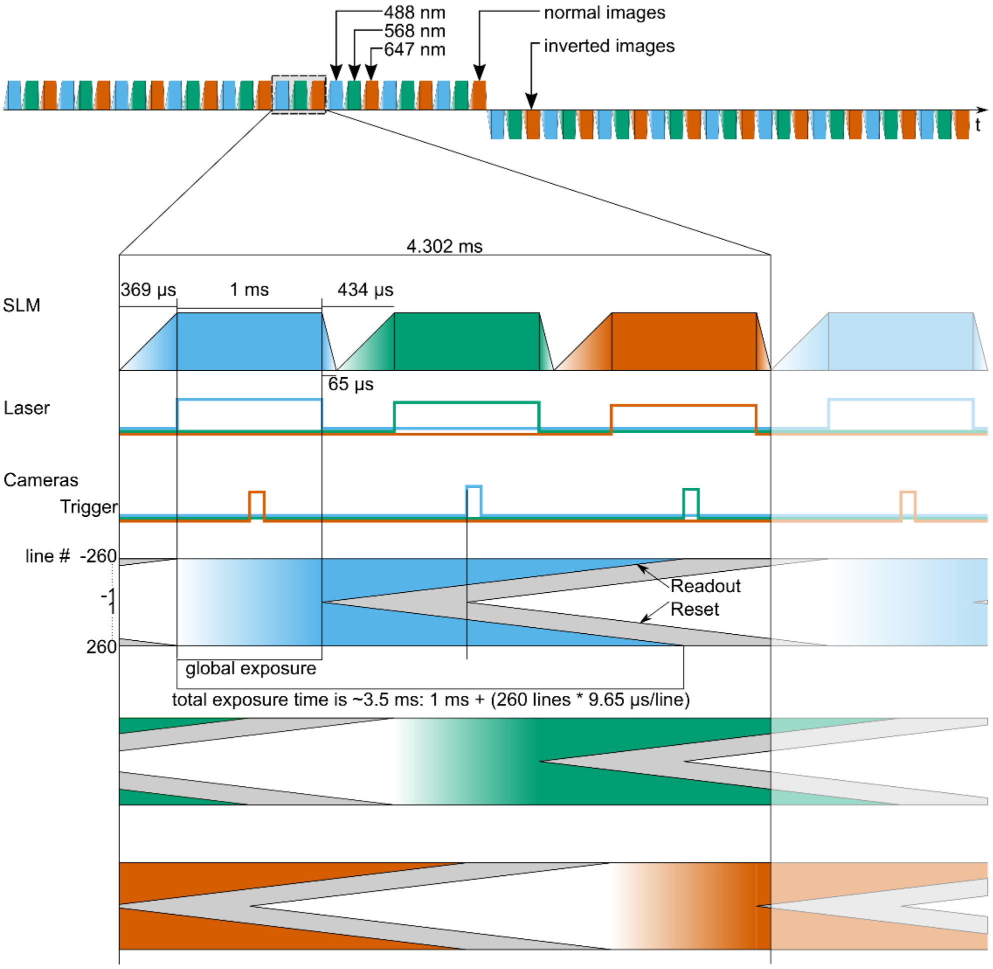

Timing schematic for the 3 color, 1 ms mode with pco.edge 4.2 cameras.

The 50 ms limit of the FLCoS-SLM is just long enough to display 3×9 individual images for 1 ms each (plus 434 µs for each switch), before the inverted have to be shown. The cameras need 9.65 µs to read two lines, symmetric around the center. The number of lines can be set in multiples of 20, so for an image with 512×512 pixels, 520 lines must be read. This takes approximately 2.5 ms.

In our timing scheme, this readout time of one camera is used to take images with the other cameras. In the 3-color mode shown here, the SLM takes slightly longer to display 3 images for 1 ms each (4.3 ms) than one camera needs to take one 1 ms image and readout the data (3.5 ms), so in this mode the switching time of the SLM is the limiting factor regarding the achievable acquisition speed. The camera trigger starts the "reset" of the sCMOS camera chip, which empties the capacitors of the individual pixels that store the collected charge (brightness information), so that each illumination starts with a "blank" chip. The cameras exposure time is set to 3.5 ms in MicroManager. With 2.5 ms of readout-time, this leads to 1 ms of global exposure, after which the readout starts automatically. The camera then waits for the next trigger to start the next reset-sequence.

The laser lines are only turned on after all 520 lines of their respective cameras have been reset and are turned off when the readout starts (global exposure). The timing is carefully adjusted on the microcontroller: A digital storage oscilloscope is used to monitor the laser and trigger signals from the microcontroller, the "trigger ready" and exposure-output signals of the cameras and the LED-enable output of the SLM. The initial timings used in the microcontroller software, which can be calculated from the graph shown above, are adjusted to the real-world conditions, with a few microseconds to spare to allow for some jitter.

Supplementary Figure 3: Structure of the real-time reconstruction software

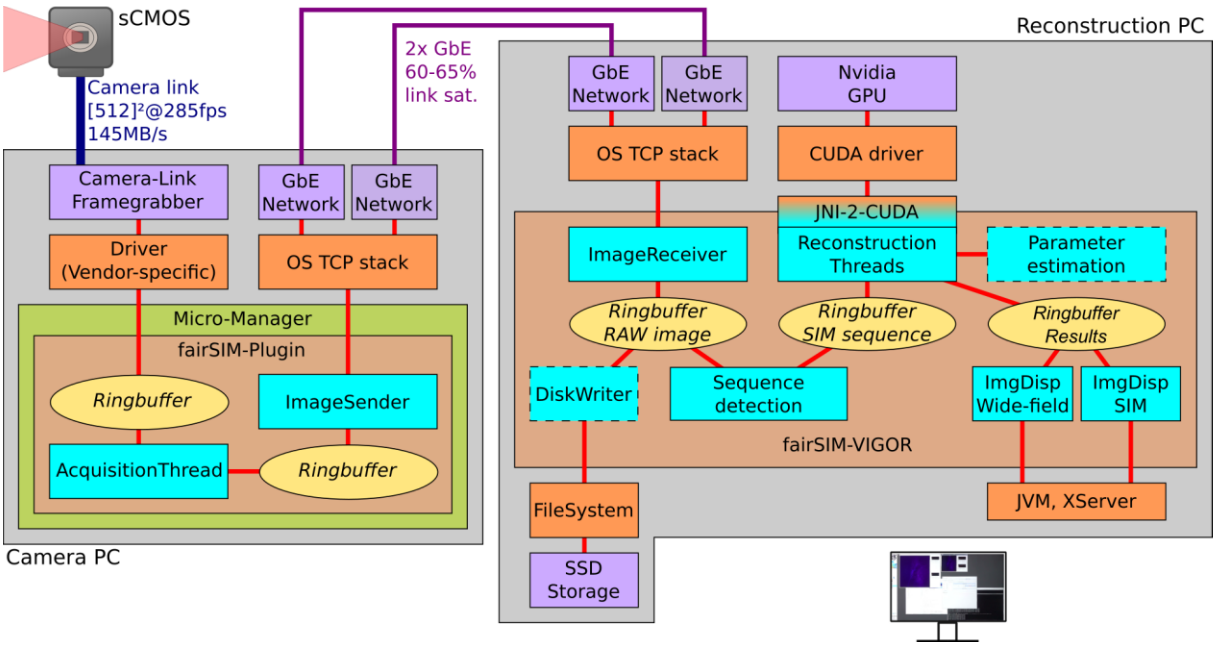

Structure of the real-time reconstruction software fairSIM-VIGOR.

These schematics show the data flow of the raw images from the cameras to the high-resolution reconstructed image. The raw images are recorded by an sCMOS camera and are forwarded via Camera Link to a frame grabber card belonging to the camera. Via the corresponding driver and device adapter, the raw images are placed in a first ring buffer of the fairSIM-Plugin running in Micro-Manager. The AcquisitionThread of the fairSIM-Plugin takes the raw images from this first buffer, adds additional meta information (channel number, sequence number & timestamp) and places them in a second ring buffer. The second ring buffer is being cleared by the ImageSender, which sends the images to the reconstruction computer via one (or more) Gigabit Ethernet TCP connections. If there is sufficient computer power, the cameras could also be connected directly to the reconstruction computer by establishing an internal network connection (“localhost”).

The image receiver of the fairSIM-VIGOR running on the reconstruction computer receives the images from the camera computer and places them in the raw image ring buffer. The Sequence detection forms complete SIM sequences from the raw data and places them in the corresponding ring buffer. Optionally, the data in the raw image ring buffer can simultaneously be written to the SSD Storage using DiskWriter, thus allowing to actually save the full raw data stream for later, offline analysis.

The SIM sequence ring buffer is being cleared by the reconstruction threads. Via the JNI (Java Native Interface), the SIM sequences are transferred to an Nvidia graphics card on which the reconstruction algorithm is then executed. If necessary, it is possible to estimate and apply new reconstruction parameters during runtime. The high-resolution reconstructed images are placed in the Results ring buffer together with the wide field image generated from the raw images, in which they are waiting to be displayed on the screen.

Supplementary Figure 4: Structure of the system control software

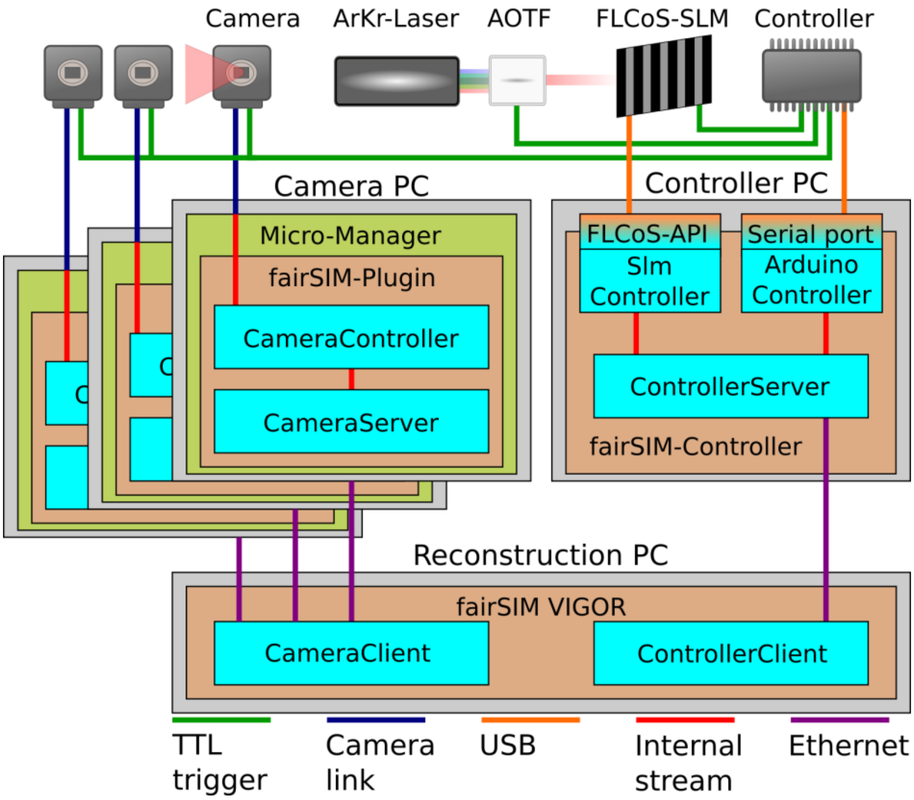

Structure of the instrument control portion of the fairSIM-VIGOR software. The entire setup is managed and run from the reconstruction computer, which in turn controls the camera computers, the SLM and the microcontroller via TCP/IP network links. The network connections required for this are implemented using abstract client and server Java classes. Network communication is based on strings and the question and answer principle, so that each command sent from the client to the server is answered, indicating the success or failure of the command to be executed. It is not necessary that the client and server are on different computers. This means that with sufficient computer performance, the entire setup can also be controlled from a single computer.

Each camera is connected to its camera computer via Camera link, on which Micro-Manager runs with the fairSIM-camera-plugin. The camera plugin includes the Java classes CameraServer and CameraController. The commands sent by the CameraClient are transmitted from the CameraServer to the CameraController, which ultimately controls the camera using the interface provided by Micro-Manager.

The FLCoS-SLM and the Arduino are connected via USB to the controller computer on which the fairSIM controller software runs. The fairSIM controller software includes the Java classes ControllerServer, SlmController and ArduinoController. The SlmController implements the Java API provided by ForthDD for the SLM. The ArduinoController uses the serial port as communication interface to the Arduino. The communication between the ArduinoController and the Arduino is also based on strings and the question and answer principle. The commands sent by the ControllerClient are forwarded from the ControllerServer to the SlmController or ArduinoController, which ultimately control the FLCoS SLM or the Arduino via the FLCoS-API or the serial port.

An ATmega328P microcontroller controls the precise timing of the setup. It triggers the cameras, the AOTF for the ArKr laser and the FLCoS SLM via digital I/O-lines, so that each individual device performs the action previously determined via fairSIM at the right time.

Supplementary Figure 5: fairSIM Easy-GUI

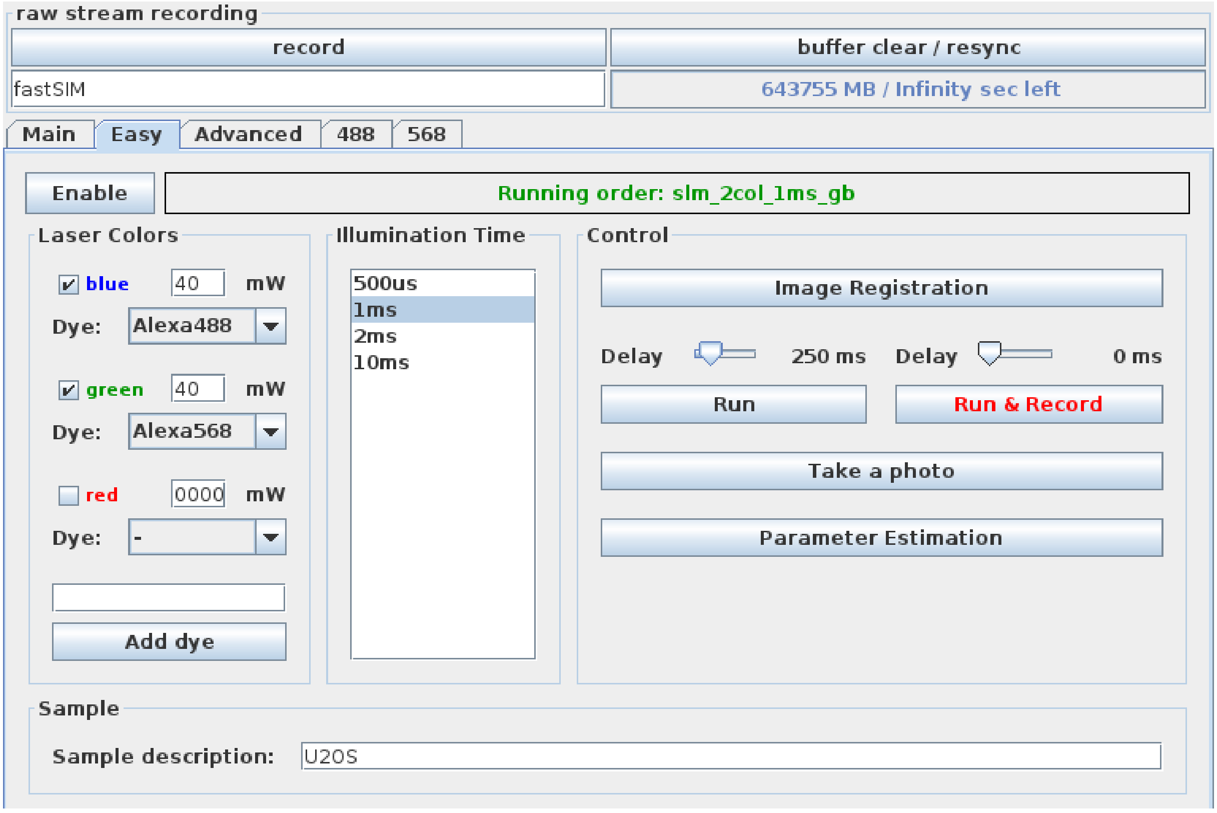

The Easy-GUI serves the user as a simple and intuitive interface for controlling the entire setup. It is closely linked in the background with the Advanced-GUI (Supplementary Figure 6). It provides automatization of all day-to-day tasks needed to operate the microscope, i.e. it configures all components (cameras, SLM, microcontroller) for the desired imaging mode, starts and stops them, and allows the user to record the image stream for later, offline analysis.

The Enable button establishes all necessary connections (SLM, Arduino and cameras). After selecting a combination of laser wavelengths, the desired exposure time can be selected. Acquisitions can then be made and optionally stored. Using the Run (or Run & Record) button, acquisitions can be started and stopped with an adjustable delay (≥ 50 ms) between the individual SIM sequences. The “Take a photo”-button is used to acquire exactly one SIM sequence. If desired, image registration and parameter estimation can be carried out using the corresponding buttons.

Entered or selected metadata (laser power, dye and sample description) are written to the image file when saving data on the storage and are available for later use along with other metadata.

Supplementary Figure 6: fairSIM Advanced-GUI

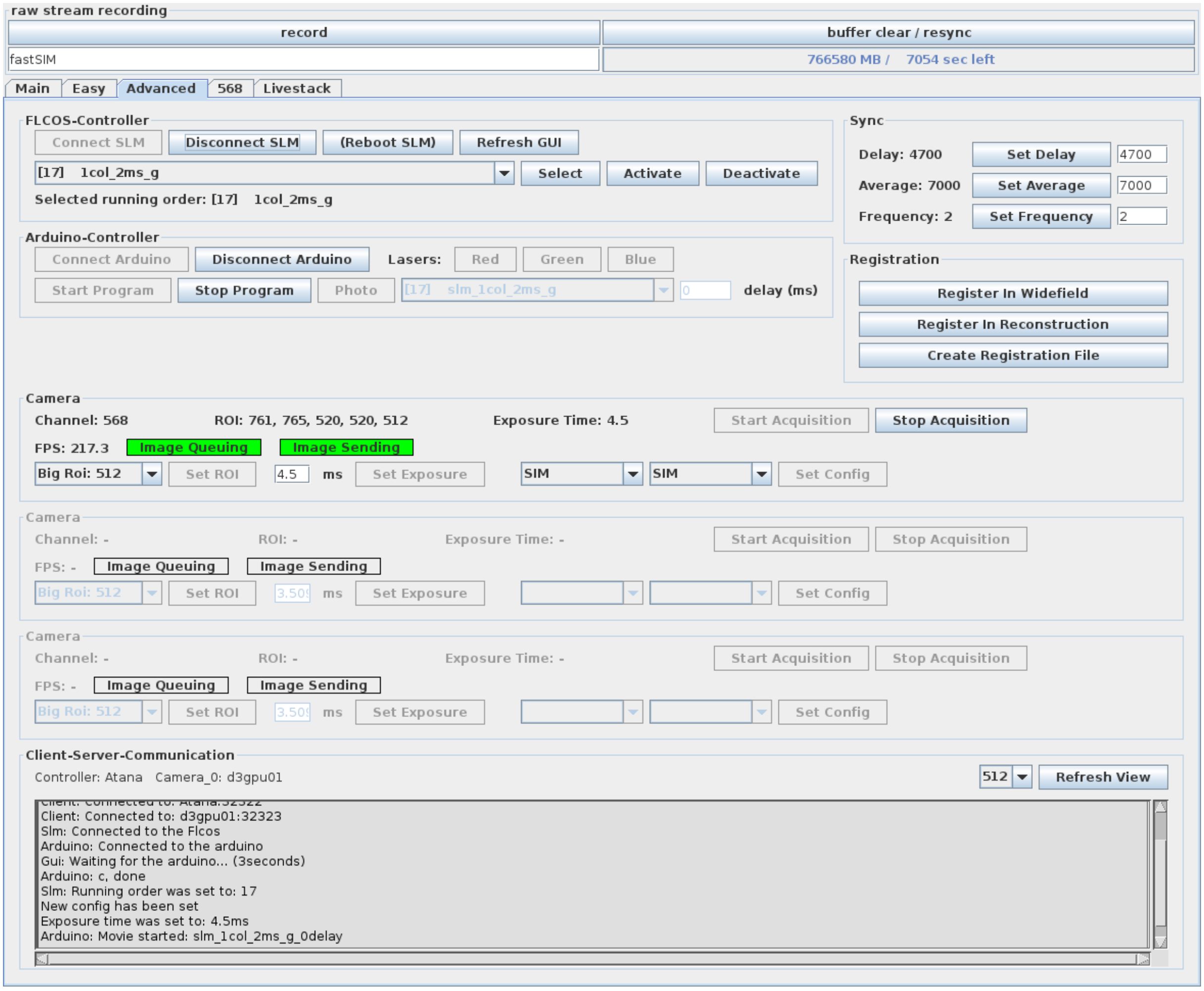

The Advanced-GUI serves as the user interface for the software described in Supplementary Figure 3 and 4 to control the individual components of the setup. Via the SLM and Arduino controller panels, the existing running orders and timing programs (see Figure 2b and Supplementary Figures 1 and 2) can be selected, started and stopped.

With the help of the three camera panels, individual settings can be made for each camera. Besides the possibility to start and stop recordings it is possible to choose between a ROI of 512×512 and 256×256 pixels. Furthermore, the exposure time and presets can be set.

The Client-Server-Communication-Panel provides the user with information about the network connections and the result of the desired actions. The "Refresh View" button reloads the display windows for the reconstructed and wide field image to adjust it for the display of a 512 or 256 ROI.

The Registration Panel can be used to register the different channels against each other. Both the reconstructed and the wide field images can be registered. To do this, a registration file must first be created, which can then be applied. The creation of the registration file uses the API of the image registration software bUnwarpJ.

Supplementary Figure 7: FluoSpheres Microspheres (0.04 µm)

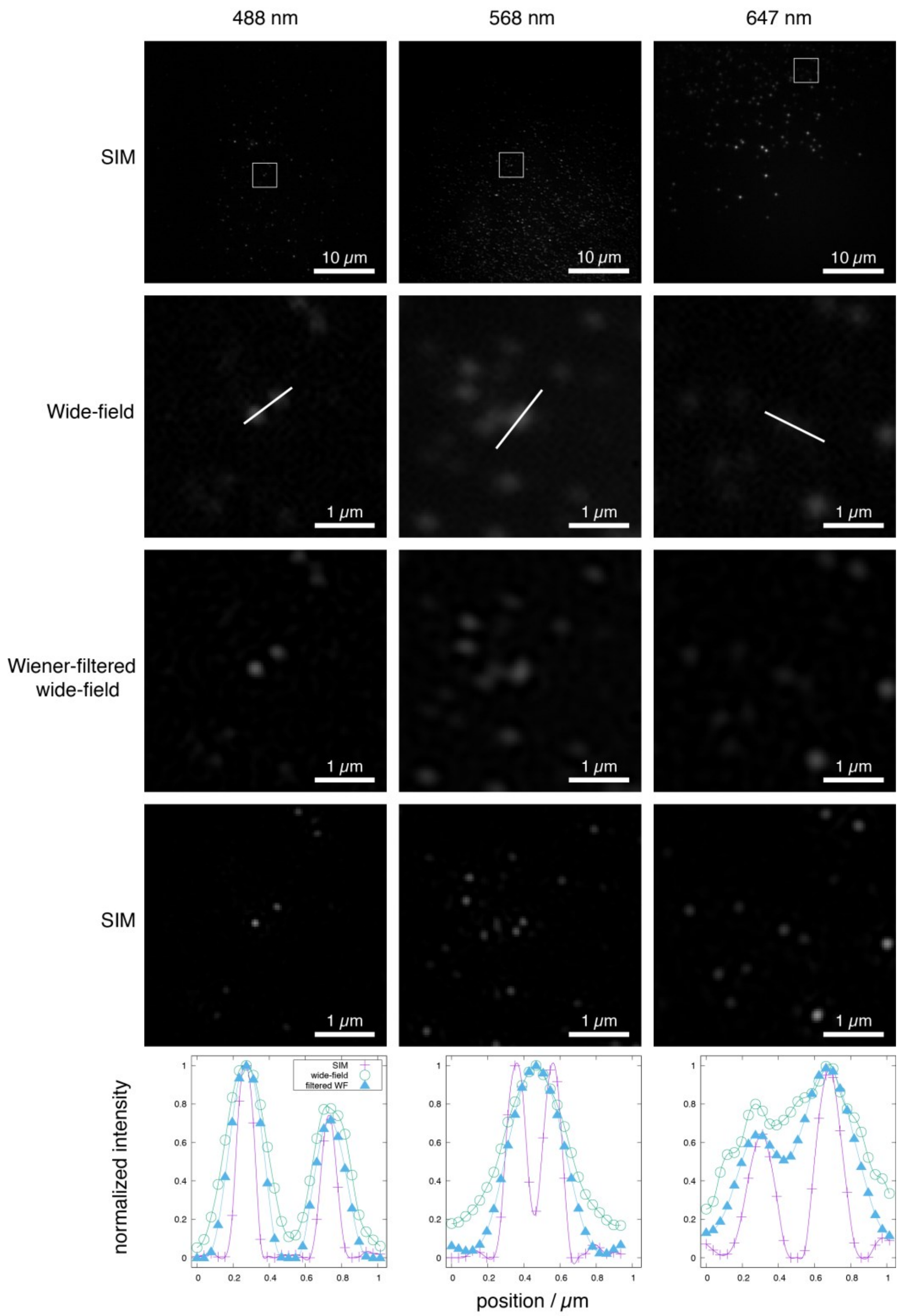

Wide-field images, SIM reconstructions and line-plots of 0.04 µm FluoSpheres™. The wide-field images were created by averaging the 9 raw SIM-frames. The SIM reconstruction shows a significant resolution improvement compared to the wide-field and the Wiener-filtered wide-field images, even with the very dim beads selected in the 647 nm-channel.

Supplementary Figure 8: Optical path

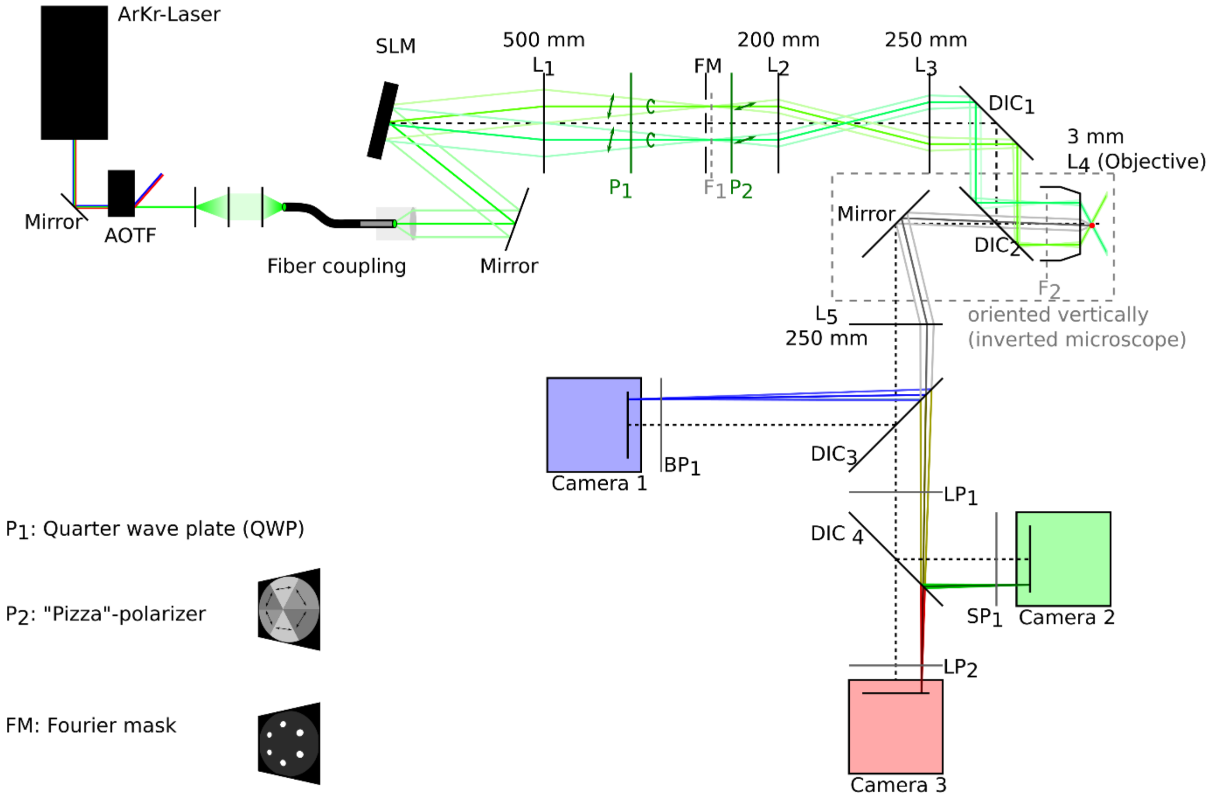

Optical path.

The laser light is controlled with an AOTF cleaned up with a single-mode fiber. It is shone onto the SLM under a small angle. The SLM reflects the light and creates a pattern in the Fourier-plane of the lens L1, where a filter mask lets the desired diffraction spots pass and blocks the other (for example zeroth and higher order) diffraction spots.

The polarization is changed from linear to circular with a quarter-wave plate. The circular polarized light is then filtered with a segmented linear polarization filter, with the polarization axis azimuthal to the optical axis ("pizza"-polarizer).

Two identical dichroic beam-splitters are used, one reflects the light horizontally by 90° (DIC1) and one reflects it vertically by 90° (DIC2). The horizontal reflection is done to compensate the polarization-change of the light when reflected by DIC2, which is a result of the different phase delay for reflected s- and p-polarized light

The detection-path uses a single tube lens (L5), long-pass dichroic beam splitters and clean-up filters in front of the cameras.

The achievable field-of-view depends on the line spacing on the SLM and its resolution: The line-spacing in the sample plane is fixed by setting a desired resolution enhancement for the SIM process, and it is linked to the SLM's line spacing through the magnification of the SLM into the sample plane. Thus, changes in magnification can be used to tweak the SLMs line spacing at constant sample-plane spacings. Due to the pixelated nature of the SLM, fine line spacings will show stronger serration, leading to a decrease in diffraction efficiency and less illumination intensity, while coarser line spacings reduce the available field-of-view. The magnification M chosen for this setup is therefore a compromise between a bigger field of view with thin lines on the SLM and a smaller FOV with thicker lines on the SLM and better diffraction efficiency.

The diffraction efficiency is approximately 5% with 488 nm and higher for longer wavelengths, as the line-thickness on the SLM scales with the wavelength.

SLMs with higher resolution could be used to illuminate a larger FOV or increase the diffraction efficiency.
