# Supplementary material for: Video-rate multi-color structured illumination microscopy with simultaneous real-time reconstruction
Source: Nat Commun. 2019 Sep 20;10:4315. doi: 10.1038/s41467-019-12165-x (PMC6754501; doi:10.1038/s41467-019-12165-x)
Supplement: Supplementary file 3 — Description of Additional Supplementary Files [file 41467_2019_12165_MOESM3_ESM.pdf]

Description of Additional Supplementary Files

File name: Supplementary Movie 1

Description: The video shows the video-rate reconstructed image acquisition of U2OS osteosarcoma cells during the operation of our 2D-SIM microscope. U2OS cells were fluorescently stained to highlight the active mitochondrial membrane (MitoTracker Green, Thermo Fisher Scientific) and the endoplasmic reticulum (ER-Tracker, Thermo Fisher Scientific). In the upper half of the video the screen of the computer that is running the GPU-enhanced SIM reconstruction software can be seen. The window on the right hand side of the computer screen shows the widefield fluorescence images of the sample, while the window on the left hand side shows the onthe-fly reconstructed SIM image of the same sample location. When the sample is moved or the focus is adjusted, the widefield view reacts immediately and the reconstructed SIM images are displayed with minimal time lag of at most 250 milliseconds. The bottom part of the video shows the two color channels to the left, as well as a composite video of both color channels to the right, displaying the same view that the operator has during the operation of the microscope.

File name: Supplementary Movie 2

Description: The video shows parts of a living U2OS cell stained with MitoTracker green (Thermo Fisher Scientific) and imaged by 2D-SIM (left) and widefield fluorescence microscopy (right). The bottom part are zoomed versions of the videos shows in the upper half. Mitochondrial dynamics was imaged with sub-second frame rates and 5 ms individual frame exposure times.

File name: Supplementary Movie 3

Description: This video demonstrates 3-color imaging of living cells and the navigation of this sample in all 3 color channels. Here, U2OS cells were stained with SYTO 9, MitoTracker Red and Tubulin Tracker Deep Red (Thermo Fisher Scientific). This experiment was conducted in a time-lapsed mode to minimize photobleaching (which is still quite noticeable in the video) using 10 ms illumination time per raw frame and a frame rate of 2.4 SIM-frames per second
